# A Preliminary Survey on Gastrointestinal Parasites of Domestic Ducks in Ahvaz, Southwest Iran

**Published:** 2018

**Authors:** Sara LARKI, Alireza ALBORZI, Rahil CHEGINI, Rezvan AMIRI

**Affiliations:** Dept. of Parasitology, Faculty of Veterinary Medicine, Shahid Chamran University of Ahvaz, Ahvaz, Iran

**Keywords:** Gastrointestinal, Parasites, Ducks, Iran

## Abstract

**Background:**

Despite ducks being birds resistant to infection, the favorable habitat of ducks such as subtropical climate or stagnant water is also a perfect place for survival of the parasites.

**Methods:**

This study was conducted from Dec 2014 to Apr 2015 to determine the prevalence of gastrointestinal parasites of domestic ducks in Ahvaz and environs, southwest of Iran. Overall, 41 fresh fecal samples were collected and prepared using formol-ether concentration, modified Ziehl-Neelsen, sheather`s floatation and zinc sulfate sedimentation methods. Light microscopic morphometry was used for identification of helminth eggs and oocysts.

**Results:**

60.97% of ducks were infected with three different nematodes and/or four protozoan parasites. The identified nematodes were *Capillaria sp.*, (50%) *Subulura* spp. (16.66%) and *Echinuria* spp. (33.33%). The protozoan oocystes were *Cryptosporidium* spp. (50%) and coccidian species (%58.33) and included *Wenionella philiplevinei*, *Tyzerria* spp. and *Isospora. mandari*. Mixed infection with two or more parasites was common. Twenty (80%) had single, four (16%) double and one (4%) triple infection.

**Conclusion:**

This is the first report of coccidian infection in domestic ducks of Iran. Further studies will be necessary on epidemiology and pathogenicity of the parasitic infections in ducks of this area.

## Introduction

Parasitic diseases as the first neglected infections involved most domestic poultry, the source of economic life of villagers, caused the loss of growth, production and overall poultry efficiency (1–3).

Chickens, turkeys, and ducks are economically the main domestic birds in rural areas. Today, the main reason for the birds keep housing ducks is due to high-quality protein meat and eggs (4–6). Moreover, other products of ducks such as down, feathers and fattened livers economically caused duck breeding in the industrial farming system (7). Moreover, rearing these birds is easier and less expensive than chickens. The population of ducks in Asia is about 937 million head while 2.7 million head of ducks living in the near east of Asia in 2005 (8) and based on unpublished statistic surveys of agriculture organization in 2015, the population of ducks in Khuzestan Province is 103784 head.

Ducks are hardy and fairly disease resistant birds, also they are known for being good scavengers outside of their nests. So, more risk factors are affected by these rural birds than industrial species.

However, this scavenger bird can be considered as final or intermediate host of many helminths or protozoan parasites, the eggs of gastrointestinal parasites are mostly passed through the feces and shed into the environment of birds and can distribute infection to human and other animals.

In addition to enteric disorders of ducks caused by intestinal parasites (9), the infected internal parasitic ducks have been seen with clinical signs of malnutrition, loss of feed conversion ratio birds and followed by decreased production and weight and even the death of birds (10), and the rural incomes are affected.

Despite the few studies on the incidence and prevalence of parasitic diseases of ducks in Iran, the present study was the first investigation of gastrointestinal parasites of domestic ducks in this area.

## Materials and Methods

Forty-one samples of duck feces were collected in different markets in Ahvaz, southwestern Iran, from Dec 2014 to Apr 2015. The ducks were marketed to sale and were housed in Ahvaz and the suburbs which comprised Mollasani, Malihekoot, Zergan, Pastorizeh, Bardieh, Seid abbas, Seddin, Sosangerd, and Abdolkhan, the residence of birds was determined by asking the owner. All ducks under this study were kept in backyard of houses and had free access to waste food and were free scavenge the outside environment at the homestead during the day. Fecal samples from ducks were collected separately and then the insulated containers were transferred to the parasitology laboratory of Veterinary Faculty of Shahid Chamran University of Ahvaz.

Fecal smears were prepared from fresh fecal samples by formol-ether concentration (11) and stained using modified Ziehl-Neelsen technique and examined under the light microscope for *Cryptosporidium* spp. oocysts (12). Three grams of each fecal sample was also emulsified in water and poured through a fine mesh sieve. The emulsion was centrifuged at 2000 rpm for 2 min and the sediment dissolved in saturated shearer's sugar solution. Test tubes were subsequently filled with the preparation, covered with coverslips, allowed to stand for 15 min and examined under the light microscope for helminthic ova and protozoan oocysts. Fecal sedimentation method using zinc sulfate solution was also carried out to detect trematode eggs. Identification of parasites was done based on the characteristics and dimensions as described (13, 14).

Positive fecal samples for oocysts were also cultured in 2.5% potassium dichromate (BDH Ltd. England) and kept in incubator (25–27°C) as described for sporulation and identification of genera and species of coccidian involved (15). Identification was based on microscopic morphometry, the number of sporozoites per sporocyst as described (13, 16). Pictures of oocysts were taken using a Dino-Lite USB microscope model AM4213U (Dino-Lite Europe) and the DinoCapture Imaging software version 2.0 of the same company.

All data obtained were analyzed using simple averages, percentages, descriptive and quantitative statistics.

## Results

A total of 41 ducks were examined, 25 cases (60.97%) were infected with one or more species of parasite and the samples of protozoan oocysts (96%) were more than gastrointestinal helminthic eggs (20%). Mixed infections with two or more species were seen in five cases (20%) while 20 fecal samples (80%) were single infection. Four samples were double infection and one sample was triple infection. In present study, no trematodes and cestodes eggs or proglottides were found.

**Fig. 1:**
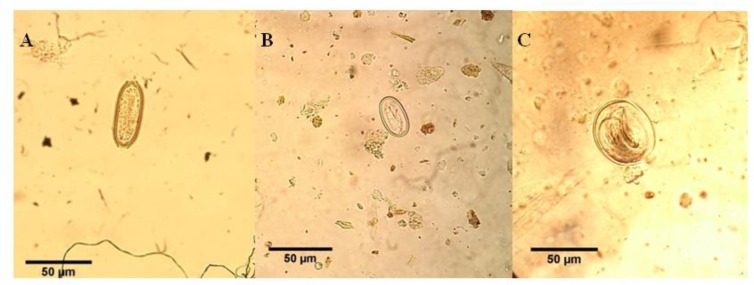
The identified species of helminthic eggs in fecal samples of domestic ducks of Ahvaz, southwestern Iran.**A:** *Capillaria* spp. **B:** *Echinuria* spp. and **C:** *Subulura* spp.

The three different species of helminths and two species of different protozoan parasites were isolated from fecal samples and identified. The diagnosed species of helminths included *Capillaria* spp. (53.2±4.5×24.3±1.8), *Subulura* spp. (52±1.4×42 ±8.4) and *Echinuria* spp. (22.5±1.4×37.5±1.7) (Fig. 1). In addition, some strongylid eggs of other animals or arthropods were found in duck feces.

The identified intestinal protozoan parasites were *Cryptosporidium* spp. (4.5±0.75 diameter) and *Coccidium* spp. (20.7±5.7×16.6±3.4) compromised *Tyzzeria* spp. (12.3±1.6×11.2±2), *Wenionella philiplevinei* (24.8±3.6×19.9±3.2) and *Isospora mandari* (22.5±0.7×21.2±0.3). Among the helminthic eggs *Capillaria* spp. was found in three (50%) fecal samples of ducks, *Echinuris* spp. was observed in two positive samples (33.33%), while *Subulura* spp*.* was found in one fecal sample of ducks (16.66%).

**Fig. 2:**
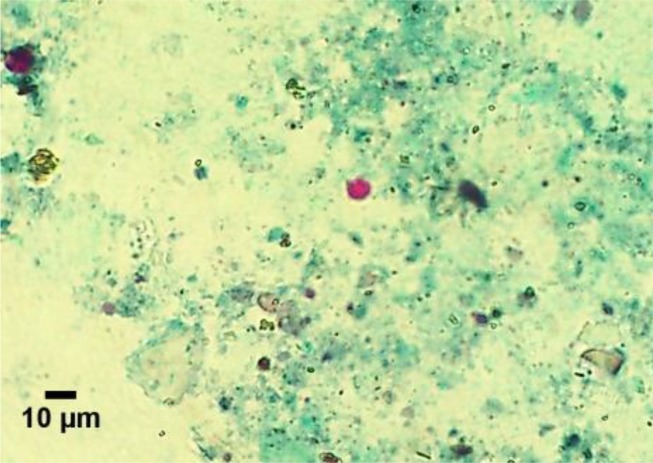
The identified *Cryptosporidium* spp. in fecal samples of domestic ducks of Ahvaz, southwestern Iran staining modified Ziehl-Neelsen technique (arrowed black) (Original figure)

**Fig. 3:**
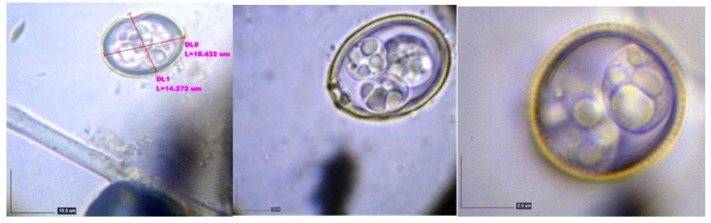
The identified Coccidial species by using a Dino-Lite USB microscope. **A:** *Tyzzeria* spp. **B:** *W. philiplevinei* **C:** *I. mandari* (Original figure)

Among intestinal protozoan parasites 14 (58.33%) fecal samples were observed, coccidial infection included *Tyzzeria* spp. (7.14%), *W. philiplevinei* (64.28%) and *I. mandari* (28.57%) and 12 samples (50%) were *Cryptosporidium* spp. positive (Fig. 2). Coccidial species were identified based on diagnostic characteristics including *W. philiplevinei,* which was the most frequently observed followed by *Tyzerria* spp*.* and *I. mandari* (Fig. 3).

Ducks which inhabit Pastoriza in the northern suburbs of Ahvaz were more infected than other regions (Table 1).

**Table 1:** Gastrointestinal parasitic infections of domestic ducks in Ahvaz, southwestern Iran and environ

***Location***	**No. sampled**	**No. positive**	***Helminths eggs No.***				***Intestinal protozoa oocytes No.***	
			***Capillaria* spp**	***Echinuris* spp.**	***Subulura* spp.**	***Tetrameres* spp**	***Cryptosporidium* spp.**	***Coccidia* spp.**
Mollasani	5	3	0	0	0	0	3	0
Malihekoot	4	3	0	0	0	0	2	2
Zergan	4	3	0	0	0	0	2	1
Pastorizeh	7	7	2	1	0	1	0	6
Bardieh	3	3	0	0	1	0	1	2
Seid abbas	6	3	1	0	0	0	2	2
Seddin	3	0	0	0	0	0	0	0
Sosangerd	5	2	0	1	0	0	0	2
Abdolkhan	2	0	0	0	0	0	0	0
Ahvaz	2	1	0	0	0	0	1	0
Total	41	25	3	2	1	1	11	15

## Discussion

The present study demonstrated that 60.97% of domestic ducks from a total of 41 birds were infected with four different eggs of helminths and oocytes of intestinal protozoa, most of the ducks were infected with single parasite (80%) and mixed infections were found in some (20%) duck stools.

There have been few studies on parasitic prevalence of domestic ducks in Iran and this investigation is the first report of gastrointestinal parasites of ducks in Ahvaz, southwestern Iran.

Helminths infections under study area were observed in 20% of ducks and identified species of helminths were *Capillaria* spp*.*, *Subulura* spp*.* and *Echinuria uncinat,* the prevalence of our findings is lower than some studies. About 46% helminths infection rate was reported in the alimentary tracts and lungs of domestic ducks in several parts of Iran that comprised *Tetrameres fissispina*, *Capillaria obsignata, C. anatis, C. contorta* (17). 70.5% of the total parasitic infection rate on gastrointestinal tracts of green-winged teal (*Anas crecca*) in Fereydunkenar in Mazandaran Province, northern Iran, *Contracaecum* spp., cestoda, *Diorchis stefanskii*, *Hypoderaeum conoideum* and *Notocotylus attenuates* were observed (18)*.* Moreover, the prevalence of infections with observation of internal organs of *Aythya nyroca* collected from central Iraq was 77.8% including *Leucocytozoon* sp*.*, *Plasmodium* sp., *Amidostomoides acutum*, *Epomidiostomum uncinatum* and *Diploposthe laevis* (19). Helminths parasite infection rate in domestic ducks in Gilan Province was 50% which consisted of *Railletina tetragona*, *Heterakis gallinarum* and *Capillaria* (20).

Only the prevalence of parasitic infection is higher than the investigations in Iraq, in the neighborhood of south western Iran; helminths infections in Native and White Peckin ducks were 5% and 11.11% respectively, consisting of *Ascaridia galli*, *Heterakis gallinarum*, *Echinoparyphium recurvatum*, *Echinoparyphium paraulum*, *Cladogynia phoeniconaiadis*, *Echinolepis carioca*, *Baerfainia anoplocephaloides* and *Entamoeba gallinarum* and *Cryptosporidium* species could not be detected in the examined ducks (21). Besides, in a study on internal organs and fecal samples of local ducks reported 47.5% infection rate that included *Hystrichis tricolor*, *Tracheophilus cymbium* and *W. philiplevinei* (3.75%) (22). The studies in Iraq due to being located in the same latitude as our study area have almost the same climatic condition that affected on parasitic distribution.

We did not observe any cestode and trematode eggs or proglottids; this may be because of lack of any habitats and optimal climatic condition of intermediate hosts of trematodes and cestodes.

The observation in Tanzania with tropical areas and in Bangladesh with a subtropical monsoon climate did not report any trematodes and cestodes in adult ducks (23, 24) while intestinal observation found 42.3% of ducks in Florida suffered endoparasitic infections that showed more than 15% trematodes and 8.9% cestodes (25). The area of this study, located in the north and central parts of the US state of Florida, is humid subtropical and south Florida has a tropical savanna climate.

In present investigation, the prevalence of parasitic infections was observed lower than other literature in Iran and most other countries. Ahvaz (latitude: 31.32°N, Longitude: 48.66°E) has a desert climate with long, very hot summers and mild, short winters. This province was considered to be a semi-arid province of Iran. Therefore, this climatic condition is not suitable for the survival and spread of helminthes parasite eggs. Moreover, climate and season can be affected by frequency and accessibility of intermediate hosts to domestic ducks (26). The gastrointestinal parasites of present study which involved domestic ducks are usually common parasites infecting domestic chicken when they are kept in the same place. Due to use of the same food and water, parasitic infections can be transmitted in some birds where they are living together.

In this investigation, the protozoan parasites were observed in 96% of ducks, 50% and 58.3% of suffered cryptosporidiosis and coccidiosis, respectively. 16.6% of domestic ducks of Gilan Province have infected with *Eimeria* oocyst which lower than present study (20).

Poor sanitation and management in habitats of birds can be followed by significant enhancement of parasitic infection risk. Moreover, contact with wild birds, insects, and rodents has an important role in the spread of sporulated oocysts mechanically (27) as in some cases the eggs of other invertebrates were seen in fecal samples. On the other hand, oocyst’s thick wall which is resistant to some disinfectants could also affect the prevalence infection rate and enable oocysts to survive in the surrounding environments for many months (28). Cryptosporidiosis rates are dependent on season, age, immunity, species of birds, environmental factors, and climate conditions which vary in different areas.

In the present study, the prevalence of cryptosporidiosis is higher than which reported 17% and 16.6% of wild waterfowl birds in lagoons of Mazandaran and Gilan Provinces of Iran were infected with *Cryptosporidium* spp. (29, 20). Moreover, this parasite in 49% of wild duck along the Rio Grande River Valley in southern New Mexico (30) while in 76% of the ducks in Brazil (31) and 57.03% of farm-raised Pekin ducklings of Bundesrepublik Deutschland (32) carried *Cryptosporidium* oocysts. Infected birds can show clinical signs and poor efficiency or no signs (32), so mild *cryptospordium* spp. infection may be as symptoms and ignored by owner. Variation in prevalence rate of different reports may be attributed to living together with other native farm animals and probability of transmission parasites compared with individual samples in some investigations.

According to our findings, the identified intestinal coccidian species were *W. philiplevinei*, *Tyzerria* spp. and *I. mandari.* This is the first report of coccidian species in domestic ducks in Iran. Compared with 21 different species of intestinal coccidiosis, only *Eimeria boschadis* and *Eimeria somateriae* are known as renal coccidiosis in ducks. *W. philiplevinei* have been found with various prevalence rates in different areas.

In present study, the prevalence of *W. philiplevinei* (64.28%) is higher than the investigations on ducks resident in Iraq (3.75%) and on Linwu ducks (14.3%) in Hunan Province of subtropical China (22, 33). *W. philiplevinei* is a considerable pathogen species of duck in China and can be effective on efficiency of products in ducks (34). Although *Tyzzeria perniciosa* is a more pathogenic species, especially in ducklings less than 7 wk of age associated with hemorrhagic enteritis (27, 35), the prevalence rate of *Tyzzeria* spp*.* was shown to have a low rate (7.14%) in ducks of present study.

Unfortunately, only a few studies have been reported on pathogenesis of coccidians in ducks. Although many species of duck coccidiosis are significant relatively nonpathogenic, some species are able to infect other birds. *I. mandari* and *E. danailovi*, a duck coccidian, were experimentally found in domestic goose (36, 37). Therefore, care should be taken seriously in duck coccidiosis. However, due to low pathogenicity of identified species of coccidiosis in infected ducks and absence of objective clinical symptoms of disease, in spite of the high prevalence of coccidiosis in feces, this infection was ignored by the owner or buyers.

This is a preliminary survey on gastrointestinal parasites of domestic ducks and further studies will be necessary on epidemiology, pathogenicity, treatment, and prevention of these parasitic infections in this area.

## Conclusion

As ducks are scavenger animals ingest a wide environmental contaminated foods, so are easily involved various species of parasites. The present status of parasitic infection in region`s ducks may cause loss of productivity, the total protein content and increase economic gain to farmers.
